# Enhancing early autism diagnosis through machine learning: Exploring raw motion data for classification

**DOI:** 10.1371/journal.pone.0302238

**Published:** 2024-04-22

**Authors:** Maria Luongo, Roberta Simeoli, Davide Marocco, Nicola Milano, Michela Ponticorvo

**Affiliations:** 1 Department of Humanistic Study, Natural and Artificial Cognition Lab, University of Naples Federico II, Naples, Italy; 2 Neapolisanit S.R.L. Research and Rehabilitation Center, Ottaviano, Naples, Italy; University of Catania, ITALY

## Abstract

In recent years, research has been demonstrating that movement analysis, utilizing machine learning methods, can be a promising aid for clinicians in supporting autism diagnostic process. Within this field of research, we aim to explore new models and delve into the detailed observation of certain features that previous literature has identified as prominent in the classification process. Our study employs a game-based tablet application to collect motor data. We use artificial neural networks to analyze raw trajectories in a "drag and drop" task. We compare a two-features model (utilizing only raw coordinates) with a four-features model (including velocities and accelerations). The aim is to assess the effectiveness of raw data analysis and determine the impact of acceleration on autism classification. Our results revealed that both models demonstrate promising accuracy in classifying motor trajectories. The four-features model consistently outperforms the two-features model, as evidenced by accuracy values (0.90 vs. 0.76). However, our findings support the potential of raw data analysis in objectively assessing motor behaviors related to autism. While the four-features model excels, the two-features model still achieves reasonable accuracy. Addressing limitations related to sample size and noise is essential for future research. Our study emphasizes the importance of integrating intelligent solutions to enhance and assist autism traditional diagnostic process and intervention, paving the way for more effective tools in assessing motor skills.

## Introduction

Autism Spectrum Disorder (ASD), is a complex and heterogeneous neurodevelopmental disorder, typically diagnosed based on the presence of difficulties in social interaction and communication, as well as the presence of restricted, repetitive, and stereotyped interests [1]. The incidence of ASD is steadily increasing worldwide, and World Health Organisation (WHO) estimates that worldwide about 1 in 100 children has autism [2].

Despite the ASD diagnosis is based on two major domains, it is well-known that autistic symptomatology is extremely heterogeneous and varies widely among individuals. This makes the diagnostic process very complex and time-consuming. Notably, the diagnosis of autism is based on subjective observations of behavior and the focus is on difficulties in communication and social interaction behavior, as well as the restricted and repetitive interests and behaviors. Many other characteristics are overlooked in the diagnostic process, considered non-central to the disorder. We refer to motor abnormalities that are commonly associated with the disorder [3–5], but have always been considered secondary symptoms and not closely related to the syndrome.

However, several literature studies demonstrated the prevalence of motor abnormalities in individuals with ASD even before the onset of language and social interaction difficulties [e.g., 6–8]. Motor disorders manifest heterogeneously, and researchers have investigated various aspects of motor skills affected by autism, such as walking [9–12], hand-eye coordination [13–15] and the reach-to-grasp movement [16,17]. Despite the relevance and presence of motor abnormalities in the clinical autistic profile currently there are no adequate tools available to accurately assess motor function in children with autism [18].

This entire line of research fits within a theoretical framework that emphasizes the idea that cognition emerges in the ongoing interaction between the individual and the environment through sensorimotor activity [19], also referring to the theory of Embodied Cognition [20]. Therefore, how an individual moves can have an impact on how they learn from the environment and, consequently, on how they explore [21,22], and on their general visuospatial abilities [23]. Results of studies investigating differences in visuospatial abilities between children with ASD and typically developing children are conflicting. Some studies identify deficits in visuospatial skills, such as fragmentation of visuospatial abilities or reduced visuospatial processing [24,25]. Other researchers have not found any differences [26], while some have even reported better visuospatial performance in children with ASD [27,28]. Nevertheless, it appears that there are differences between the groups regarding this aspect, and further investigation is warranted.

In the meantime, researchers are delving deeper into the analysis of differences related to movement. The recent emphasis on movement has highlighted the need to identify an ecologically valid tool to measure this behavior, and researchers have been moving towards the development of game-based tasks using smart-tablet devices. For instance, Perochon et al., in the 2023 [29], introduced a bubble popping game on a tablet to assess visuo-motor skills in a group of typically developing children and a group with ASD, demonstrating differences between the two groups in the pace of task performance and finger placement [29].

In a context where autism assessments can become more objective and generate large amount of data, it is crucial to identify a reliable method to analyze these data. Thus, to address these challenges and improve the timeliness and reliability of diagnosis, it has become essential to explore intelligent solutions. Indeed, improving the efficiency, scalability, objectivity, and reliability of measures of autism-associated behaviors also holds significant promise for improving early screening and intervention efforts for the disorder. With the availability of vast amounts of data collected through new technologies such as apps, tablets, and other devices that enable performance recording, the use of machine learning (ML) methods appears to be a fitting and relevant choice.

Some researchers have moved in this direction, for instance, Simeoli et al., conducted a comparative analysis of motor trajectories in children with autism and typically developing children. The objective was to validate the hypothesis that children with autism display unique motor patterns distinct from those observed in typically developing (TD) children. They used an artificial neural network (ANN) that achieved 93% of accuracy in classifying ASD and TD [30]. However, it was difficult to say how the two groups differed. For this reason, in line with this study, Milano et al., in 2023 [31] conducted another study, on the same data to deeply explore the power of each feature for the classification. Their results revealed that maximum acceleration, minimum acceleration, standard speed, and standard acceleration play a significant role in the classification process [31].

Therefore, using these algorithms may enable motion pattern recognition and ASD classification regardless of the nature of the data. The potential of these analyses, in addition to increasing knowledge about the syndrome, can also assist in making more informed decisions regarding the treatment and medical condition management [32,33]. This research study aims to develop and test an innovative approach for collecting and analyzing motor data in an ecological and child-friendly setting.

Specifically, we explore the effectiveness of using a custom-built serious game for movement analysis to assist in understanding the motor patterns of ASD. Furthermore, we aim to compare different ANN models, developed on raw data to explore their potential in enhancing classification. By comparing the performance of two neural models on a raw and a feature-enriched datasets, the hypothesis seeks to identify which approach is more effective in motor data analysis. Finally, through experimenting with the generation of new trajectories by modifying the features selected from the original trajectories, we assessed the neural models’ ability to adapt to variations in motor data and explore the specific feature’s role.

Overall, this research hypothesis can be valuable for developing more effective tools for assessing motor skills in children and advancing the understanding of how neural networks can contribute to this process.

## Methods

### Participants

Twenty children between the ages of 3 and 6 participated in the study. Participant recruitment began on February 13, 2023, and concluded on March 21, 2023. The sample consisted of two groups: children with a diagnosis of autism (ASD) and typically developing (TD) children.

The ASD group comprised 10 children (mean age: 4 years ± 0.94 SD) diagnosed with ASD. The TD group included 10 typically developing children (mean age: 3 years and 9 months ± 0.1SD).

Participants in the ASD group were recruited from the Neapolisanit S.R.L. Rehabilitation Center, where they were undergoing psychomotor and speech therapy treatments. Inclusion criteria for the ASD group consisted of a clinical diagnosis of autism, an age range between 3 and 6 years, and the absence of comorbid conditions. All ASD diagnoses were conducted using the Autism Diagnostic Observation Schedule (ADOS-2) [34] by healthcare professionals and industry experts who were independent of our research laboratory and study.

The ASD group comprised children across a range of symptom severity levels within the spectrum, all of whom were able to engage with a tablet, suggesting a moderate level of functioning. TD participants were recruited from an educational institute including pre-primary and primary school grades in the province of Naples. TD participants were recruited from various classes and without specific assessment of their pre-existing abilities. The only criterion for their selection was the absence reported by parents and teachers of neurodevelopmental disorders. Before the experimentation, parents of all the participants read and signed the informed consent, guaranteeing their full understanding and consent regarding their child’s participation in the research. The experimental protocols used in the study received approval from the Ethical Committee of Psychological Research of the Department of Humanities of the University of Naples Federico II.

### Materials

**Fig 1:**
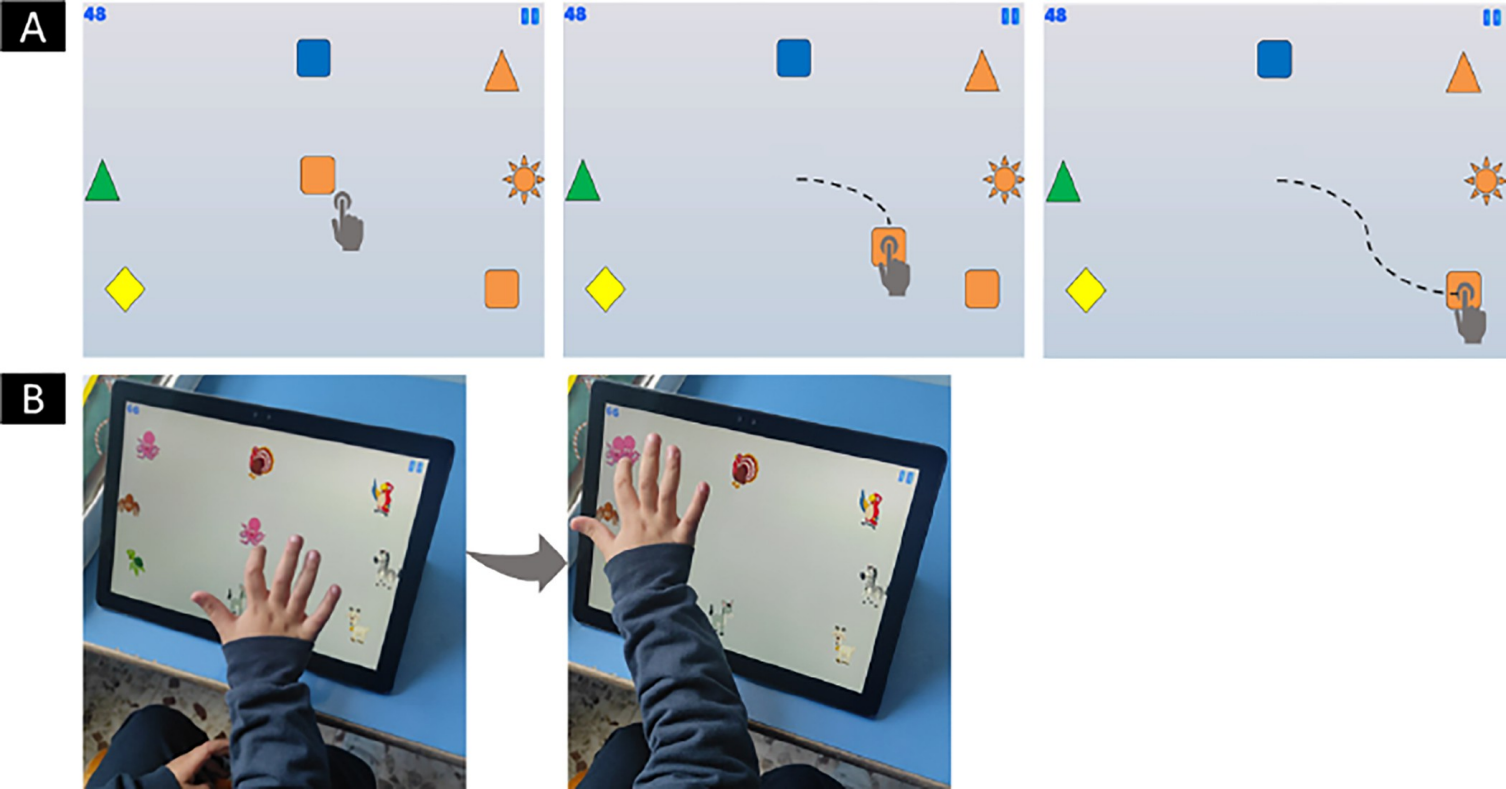
Example of a played game scene. **(A)** An example of drag-and-drop action while playing the game. Users must drag the image placed in the center of the screen onto the target, avoiding distractors. The dotted line represents the trajectory drawn by the user. **(B)** A user’s correct execution on a game scene where the image of an "octopus" in the center of the screen has been dragged to its target avoiding the 7 distractors.

The game software was developed using Unity 2D and C#, specifically for tablet devices. The game involves a drag and drop task. Specifically, the user has to drag an image placed in the center of the screen, with coordinate (0,0), to a target image, placed in one of eight positions with angular distances of 45 degrees, as in Fig 1A [35].

**Fig 2:**
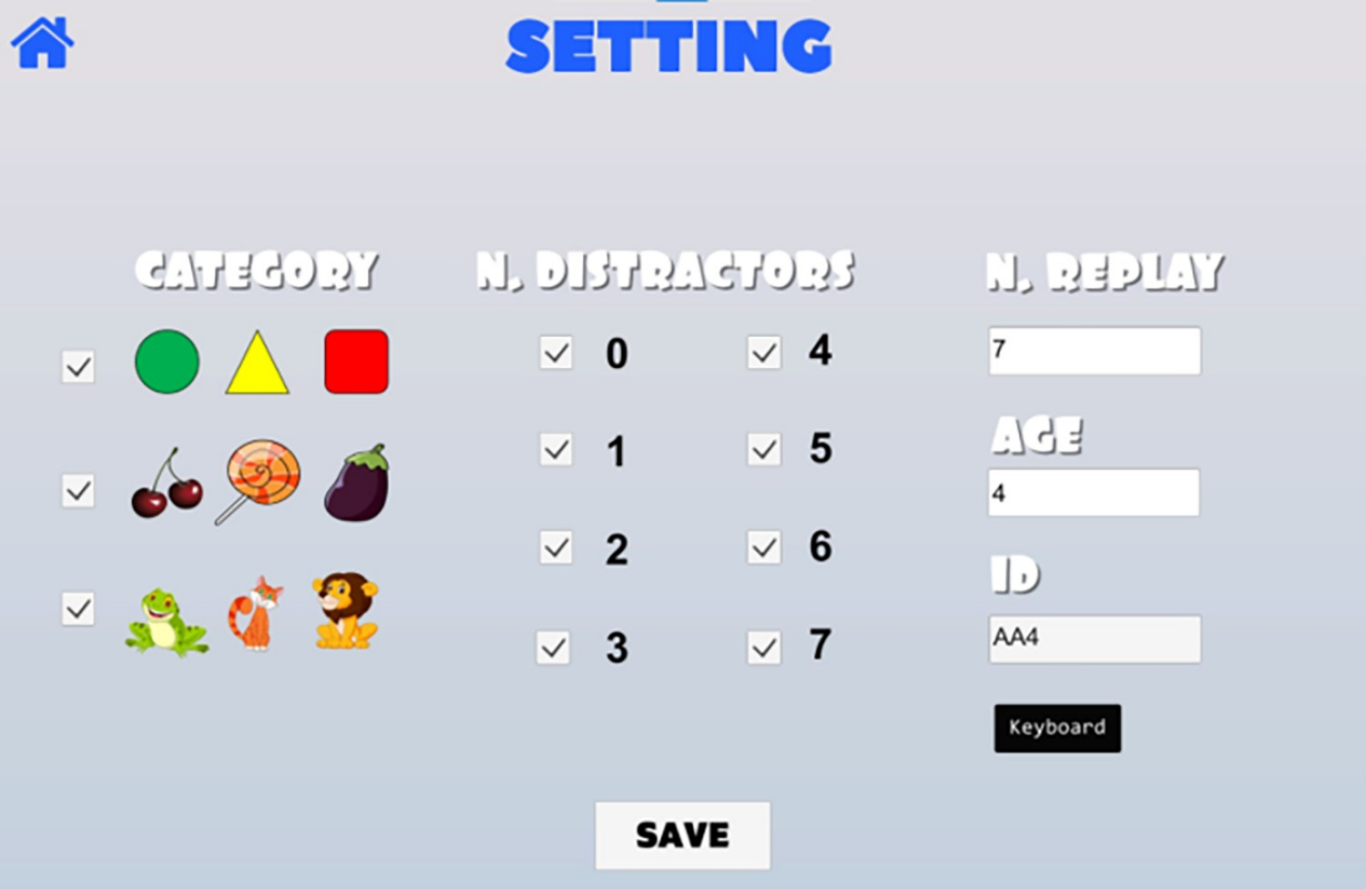
Layout of the customization settings section. The page displays the options available to the experimenter from left to right: (i) the 3 semantic category options (animals, fruits, and geometric shapes) headered "category"; (ii) The number of distractors that can affect the game’s complexity, “n. distractors"; (iii) The number of times the same arrangement of image category and number of distractors can appear, "n. replay"; (iv) the section where the child’s age can be entered "Age"; (v) user identification code "ID".

The software includes a "setting" section to customize the game scenes and adapt them to the child’s skill level (Fig 2). Through the setting section the experimenter can customize the game features, such as the image category, between geometric shapes, fruits, and animals, the amount of game scenes, and the number of distractors.

In this study, the same setting conditions were used for all the participants.

### Experimental protocol

During the study, participants were seated at a table with height ranging from 45 to 60 cm, depending on the age of the child. The experimenter was sited on the right side of the child, at about 30 cm. The task was performed on a 12.3-inch Dell Latitude tablet placed on the table in front of the child (Fig 1B). To ensure a full view of the game, the tablet was raised and tilted at an angle of about 60° to the table surface. The examiner did not provide specific instructions on how to play the game, but the child was encouraged to interact with the tablet independently, giving him the opportunity to understand the logic of the game. Each child participated in a single gameplay session. Despite the same game settings for all children, involving a fixed number of gameplay scenes with an increasing number of distractor images, the time taken to complete the entire task differed from one child to another.

However, two types of “booster” were provided while playing the game. Firstly, a video tutorial starts when the child presses the "play" button. Secondly, the image to be moved vibrates, grabbing the user’s attention. The video tutorial was necessary to avoid stressful social situations and improve engagement and autonomy.

### Data acquisition

The game software records the x and y coordinates of the dragging movements performed by the child. The recording begins when the subject starts dragging the image to be moved positioned in the center of the screen, while any touches or drags that occur outside the image to be dragged are not recorded. Furthermore, trajectories that start from the moving image but stop without reaching the target, also were excluded from recording. The data is saved at a frequency of 20 Hz, namely, an amount of 20 coordinates per second were recorded.

Data acquisition occurred throughout the entire game’s execution period.

Although we had set the recording to specific trajectories which complete the dragging from target to target, these trajectories could vary significantly in length (number of coordinates), depending on how the user approached the task. Therefore, to make the trajectories comparable, we only used trajectories with lengths ranging from 20 to 69. A trajectory with a length of 20 indicates that it comprises 20 distinct points, each defined by x and y coordinates.

Asynchronous programming was implemented to facilitate data storage and maintain optimal system performance. Tracking data has been recorded, such as the correctness of the trajectory, the vector distance between the image to be dragged and the target, and the x, y coordinates of movement in time and space of finger dragging movement, and these last has been used for the analysis.

### Machine learning model

ML employs specialized algorithms to extract information from large datasets to make informed decisions about new data [36]. ML models require an intensive training period on a large dataset known as the "training dataset." After training, the model must be tested to assess its accuracy and ability to generalize to new, unknown data, referred to as "test datasets”.

ANNs are one of the main techniques used within ML, it is a computational model composed of artificial "neurons", which is inspired by the functioning model of the brain. These networks are composed of layers of interconnected artificial neurons that process information through a learning process. Each neuron receives input, performs an operation, and transmits the output to subsequent layers. Learning occurs by changing the weights of connections between neurons in response to training data, allowing neural networks to recognize complex patterns and perform prediction or classification tasks. Therefore, they can help detect the presence of specific clusters.

### Feature extraction and data preprocessing

The features were derived from consecutive sets of raw coordinates. The x and y coordinates were grouped into trajectories. The raw coordinates were rotated [37]. To perform the rotation, the angle between two points with respect to the origin was calculated (1).


$$\Delta x=x_{2}-x_{1}$$ (1)

$$\Delta y=y_{2}-y_{1}$$

θ=atan2(Δy,Δx)

Where *Δx* represents the difference between the x coordinates of the second point and the first point; *Δy* represents the difference between the y coordinates of the second point and the first point; *θ* represents the resulting angle between the two points calculated using the atan2 function.

Generally, the function *atan*2(*Δy*, *Δx*) returns an angle between -π and +π radians, but angle *θ* was represented in a range from 0 to 360 degrees (i.e., from 0 to 2π radians). When angle was negative (i.e., less than 0), a value of 360 degrees was added to move the angle in the range from 0 to 360 degrees. In this way, any angle calculated was positive, representing the angle between the coordinates with respect to the origin of the coordinate system (2).


ϕ=θifθ≥0 (2)

ϕ=θ+360°ifθ<0

Where θ is the original angle obtained from the atan2 function; ϕ is the transformed angle within the range from 0 to 360 degrees.

After calculating the angle between the two points, we apply the cosine and sine of this angle to obtain the new coordinates of the point (3).


$$x^{\prime }=x\;cos\;(\theta )-y\;sin\;(\theta )$$ (3)

$$y^{\prime }=x\;sin\;(\theta )+y\;cos\;(\theta )$$

Where (*x*′,*y*′) are the new coordinates of the point after rotation; *x* and *y* are the original coordinates of the point; *θ* is the angle of rotation in radians; *cos* (*θ*) represents the cosine of the angle θ; *sin* (*θ*) represents the sine of the angle θ.

**Fig 3:**
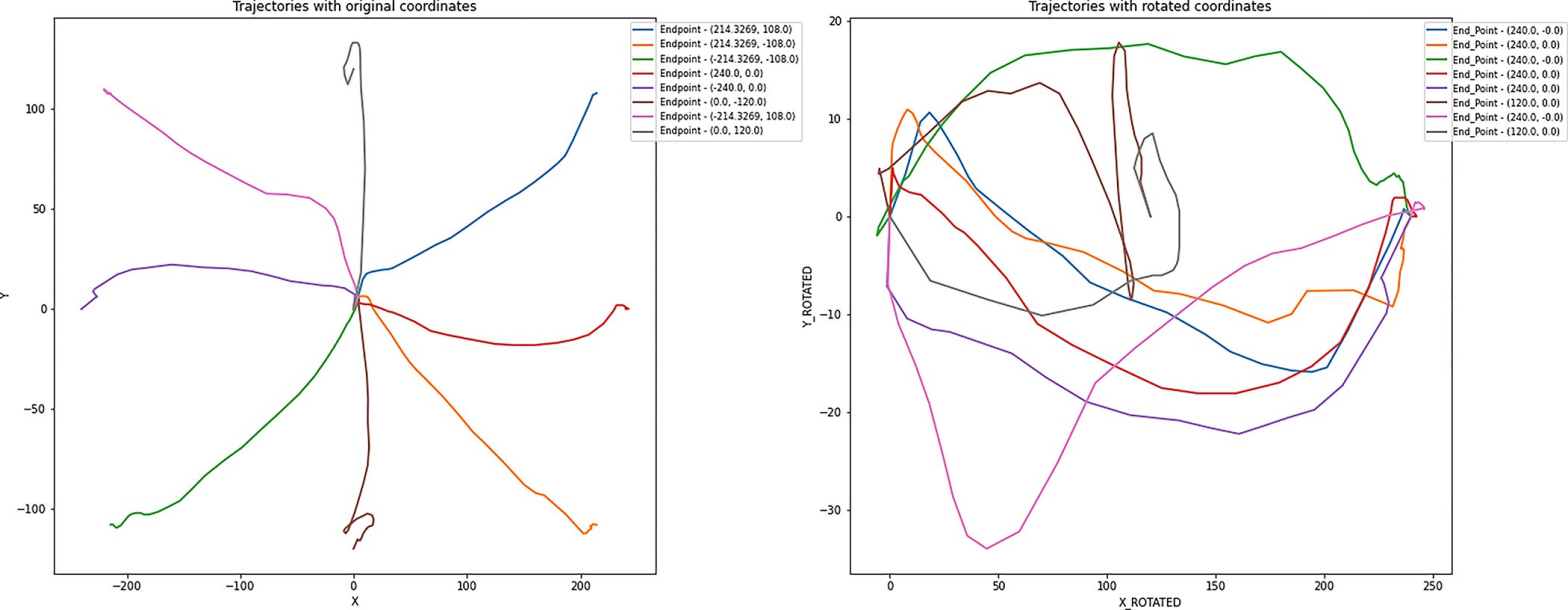
Trajectories graphical representation. Graphical representation of the original trajectories and their respective clockwise rotated trajectories.

This process allows the point to be rotated around the origin, resulting in its new coordinates after rotation. In Fig 3, the original trajectories and their rotated counterparts can be observed graphically. As can be seen from the image, the original trajectories have the following endpoints: (0, 120), (0, -120), (240, 0), (-240, 0), (214.3269, 108), (214.3269, -108), (-214.3269, -108), (-214.3269,108), which correspond to the 8 possible positions of the target or distractor image. The trajectories have been rotated clockwise so that their start and end are aligned with the x-axis. Trajectories ending at (240,0) remain unrotated.

To illustrate the rotation process in detail, we considered a specific example where the endpoint of a trajectory is at (-214.3269,108). The goal is to rotate this trajectory so that its endpoint aligns with the x-axis (240,0).

The first step is to calculate the rotation angle θ required. Assuming that the initial point of the trajectory is the origin (0,0) and the endpoint is (-214.3269,108), we can use (1) to calculate rotation angle. For this trajectory, θ ≈154.75. Once we have calculated the angle, each point of the trajectory is rotated by this angle in a clockwise direction. Each point of the trajectory was transformed with the calculated angle θ, effectively aligning the endpoint with the x-axis (2).

After the rotation of the coordinates, a standardization process was applied to the data. Standardization involves scaling the data to have zero mean and unit variance, ensuring that all features are on a similar scale. This step helps to remove any potential bias or variations in the data that could affect the model’s performance. After standardization, the trajectories were subjected to a padding technique to address the issue of different lengths. Padding involves adding zeros to the trajectories, ensuring that all sequences have the same length, as the model requires fixed-length inputs. By the means of padding technique, the model can effectively handle sequences of varying lengths and process them consistently [38]. Only trajectories with a length between 20 and 69 were considered for data analysis. The dataset used in this study consisted of a total of 738 samples collected from 20 subjects (309 samples for TD and 429 for ASD). A one-hot encoding of the target variable facilitated the classification process by representing the distinct classes in a categorical format.

TD Individuals were assigned the label (1,0), while ASD individuals were assigned the label (0,1). Once the data was standardized and padded, the compiled datasets, consisting of aggregated and rotated trajectories, were then used as the input for two different ANNs.

The first neural model takes only two input features, namely the X and Y coordinates of the trajectories. This means that the model exclusively deals with the spatial position of the data. On the other hand, the second neural model takes four different features as input: the X and Y coordinates and the point-to-point velocity and acceleration of the trajectories. This model is designed to capture not only the spatial position but also the dynamic information of the data, such as velocity and acceleration. In the case of the model that considers the four features, we needed to address the management of missing data. This need arose because the calculation of velocity and acceleration involves comparing consecutive points along trajectories. To adequately prepare the data for standardization, the missing data, pertaining to the values of acceleration and velocity for the first point in each trajectory, were replaced with zeros.

### Classification methods

**Fig 4:**
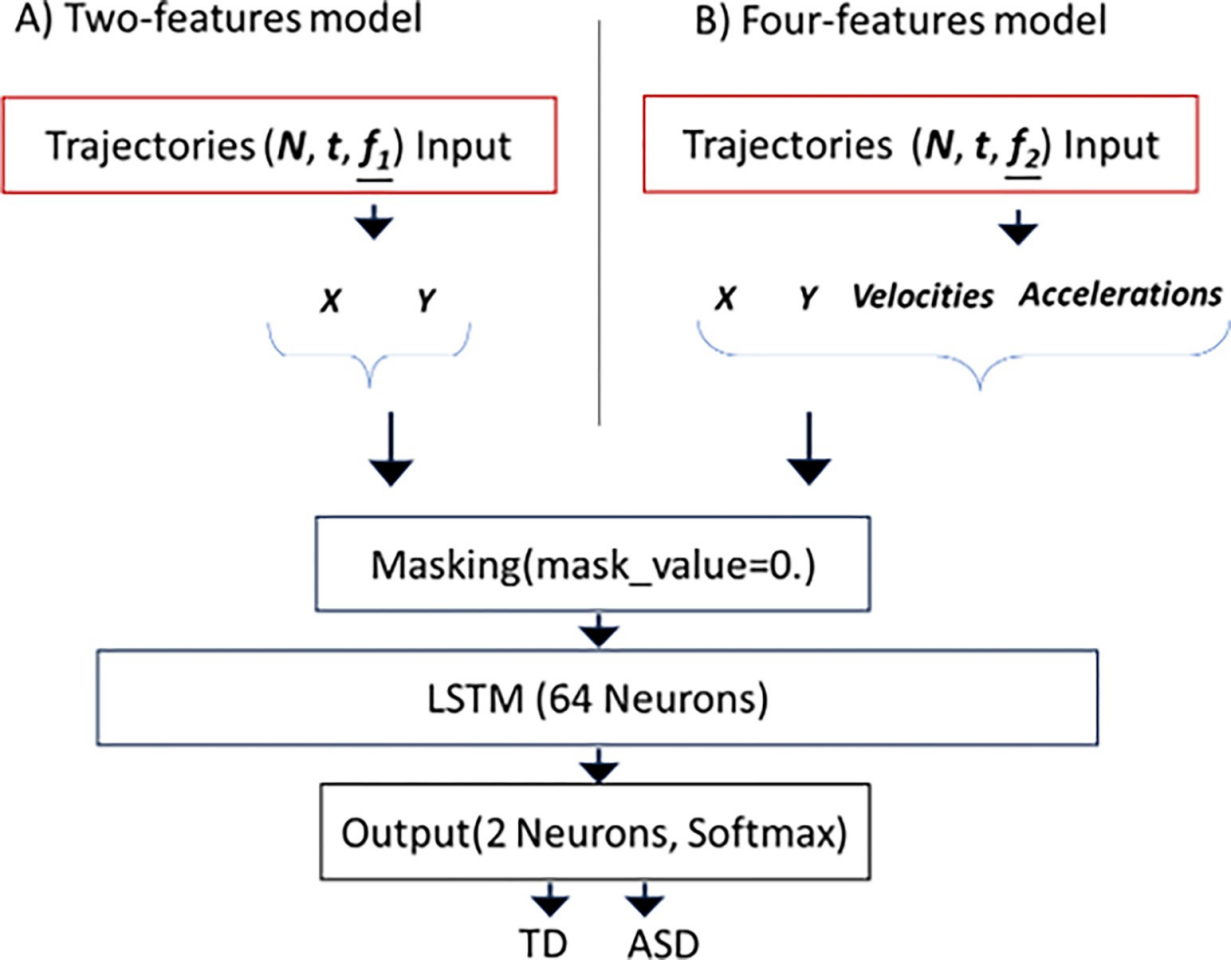
Graphic representation of the two models. **(A)** For the two-features model’s architecture, N represents the number of samples, effectively representing the trajectories as input, t denotes the timestep, and the features f_1 are aligned with spatial coordinates, specifically the x and y positions. **(B)** For the four-features model’s architecture, N also represents the number of samples, indicating the trajectories as input, t indicates the timestep. However, the features f_2 are: The spatial coordinates x and y positions, velocities and accelerations. The network architecture for both models remains the same, comprising a masking layer to ignore "0" values introduced by padding. A LSTM layer, a type of recurrent network with 64 units. The final layer is a Dense output layer equipped with a softmax activation function and two neurons for the two classes, TD and ASD.

The two models were created to classify children’s trajectories as either belonging to autistic or typically developing children based on the input sequences. As mentioned previously, the input sequences represent trajectories and consist of 69-time steps, each containing two features, for the first model. While, in the second model we have as input, trajectories with four features, x and y coordinates, velocity and acceleration (Fig 4). The selection of 69 time steps was a methodological decision informed by preliminary data analysis and model performance evaluation. In the initial stages of our study, we observed that using the entire trajectories for analysis introduced a significant amount of noise into the data. This noise adversely affected the model’s ability to generalize and extract relevant features for classification purposes.

Upon experimenting with different lengths of time steps, we found that a threshold of 69 time steps minimized data noise and improved model performance by facilitating more accurate feature extraction and enhancing the model’s classification capabilities. Additionally, our decision to select 69 time steps was significantly influenced by our intention to emphasize the initial movement within the trajectories.

For both models, the network architecture is the same. The only difference is the shape of input data (Fig 4). To handle variable-length sequences, a Masking layer is introduced at the beginning of the model. The Masking layer identifies and masks any 0 values in the input sequences by setting them to a specific mask value (in this case, 0). This masking process ensures that the model ignores the padded or irrelevant parts of the sequences during training and focuses only on the relevant parts of the trajectories, effectively handling sequences of different lengths. After the Masking layer, there is a Long Short-Term Memory (LSTM) layer with 64 neurons. The LSTM unit is a type of recurrent neural network that is effective in analyzing temporal sequences. This layer of LSTM is responsible for capturing relevant patterns and features in trajectory data [39]. The last layer is a fully connected layer with 2 units, corresponding to the two classes of classification: TD and ASD. The softmax activation function is employed to compute class probabilities, ensuring the output values represent the likelihood of each class (4).


$$\sigma (z_{i})=\frac{e^{z_{i}}}{\sum_{j=1}^{K}e^{z_{j}}}\;for\;i=1,2,\ldots ,K$$ (4)

Where σ(z_i) represents the probability that the input belongs to class i; z_i is the value associated with class i; ∑_(j = 1)^K e^(z_j) is the sum of the exponential scores of all possible classes.

The model is trained using Adaptive moment estimation (Adam) learning algorithm, a popular choice for gradient-based optimization in deep learning [40]. A learning rate of 0.001 is set to control the step size during weight updates. The objective of the training process is to minimize the loss. By minimizing this loss, the model maximizes its ability to accurately classify trajectories.

To evaluate the model’s performance, the categorical cross-entropy loss (CE) and accuracy metric are employed (5). The loss quantifies the dissimilarity between predicted and true labels, while accuracy measures the proportion of correctly classified instances.


$$CE=-\sum_{c=1}^{M}y_{o,c}log\;(p_{o,c})$$ (5)

Where M is the total number of classes; y_(o,c) represents the ground truth (label) for instance o and class c. It is 1 if the instance belongs to class c, otherwise 0; p_(o,c) represents the probability predicted by the model that the instance o belongs to class c. We selected the ’binary accuracy’ metric for its widespread application in binary classification scenarios, where it quantifies how often predictions align with the actual labels.

To evaluate the predictive ability of the two models in classifying motor trajectories, we used k-fold cross-validation. Given the relatively small size of our dataset, we opted for a four-fold cross-validation approach. The validation test consists of 110 samples, precisely 15% of the entire dataset (738).

### Simulated trajectories from existing data

One of the objectives of the study was to examine whether the average acceleration of trajectories could be a significant factor in influencing the prediction and classification of autistic versus typically developing children. To do so, we tested both the models (two-features model and four-features model) on simulated trajectories. Specifically, we performed computational variations on the acceleration values to explore how such variations could impact the classification process.

To thi end, new trajectories were created by selectively retaining a variable number of points within each existing trajectory. This selective removal or preservation of points within the trajectories allowed us to manipulate the average acceleration of the trajectories. The process of trajectory simplification involved reducing the number of points within a trajectory while maintaining an equidistant distribution among the remaining points. To obtain an equidistant distribution, the indices of the points to be maintained uniformly along the original trajectory were calculated, thus creating a simplified version of the trajectory (6).


M=N⋅p

$$Indices(i)=\frac{i}{\left(M-1)(N-1\right)}$$ (6)

Where Indices(i)represents the index of the point in the simplified trajectory at position I; N is the total number of points in the original trajectory; M is the desired number of points in the simplified trajectory; p represents the specified percentage; i varies from 0 to M-1 to determine the equidistant indices.

**Fig 5:**
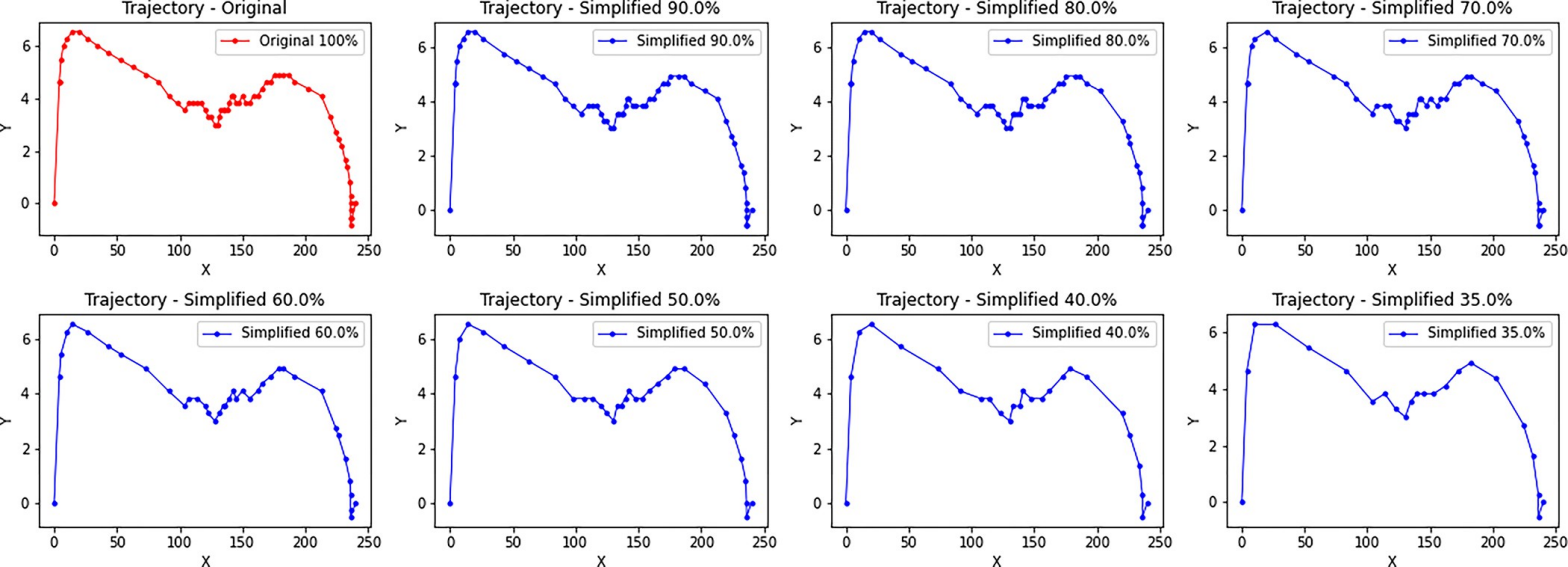
Graphical representation of trajectories simulated from a pre-existing trajectory. The figure illustrates how a trajectory undergoes changes while maintaining different percentages of its points: 90%, 80%, 70%, 60%, 50%, 40% and 35%.

It is also fair to clarify that if the original trajectory had a positive average acceleration, removing points would result in an increase in average acceleration. In contrast, if the original trajectory had a negative average acceleration, removing points would lead to a further decrease in average acceleration. The decision to set the 35% threshold as the minimum for trajectory simplification was guided by a careful balance between minimizing data complexity and preserving essential information within the trajectories. It was identified that trajectories retaining less than 35% of their original points typically comprised fewer than 20 points. This count is crucial for maintaining the integrity of key trajectory aspects, such as the overall shape and directional changes, essential for analysis accuracy and model predictions. In selecting simplification percentages, ranges were chosen to create a marked distinction between simplified trajectories, thereby reducing the risk of overfitting. Initial analyses showed that minor increments, like 5%, led to overly similar trajectories, undermining the model’s capacity to generalize to new data. Preliminary proofs indicated that smaller increments, such as 5%, resulted in trajectories that were too similar to each other, diminishing the model’s effectiveness in generalizing to unseen data. Therefore, intervals ensuring adequate variability among simplified trajectories were selected, enhancing the model’s robustness and generalization abilities. Specifically, we examined different simplification rates, including 35%, 40%, 50%, 60%, 70%, 80% and 90%. To properly test the model, all generated trajectories that had less than 20 points were excluded (Fig 5).

## Results

**Fig 6:**
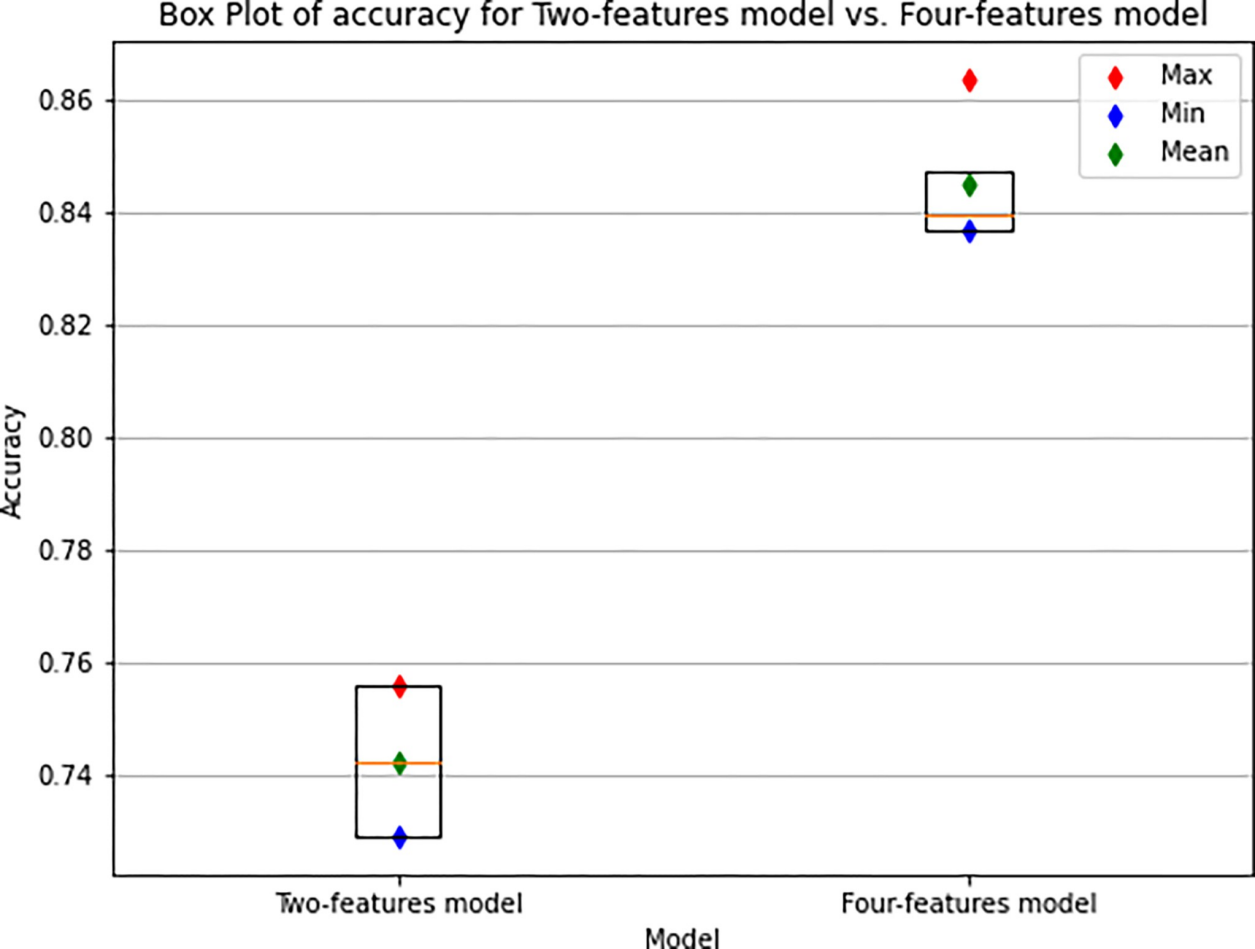
Box Plot of the accuracy of the two-features model and four-features model within the 4 folds. Each box displays the distribution of model accuracy, with the horizontal line inside it representing the median (the central accuracy value). The upper and lower quartiles of accuracy are depicted as the upper and lower boundaries of the boxes. Additionally, the mean accuracy values, the maximum and minimum accuracy values for each model are also clearly indicated.

**Fig 7:**
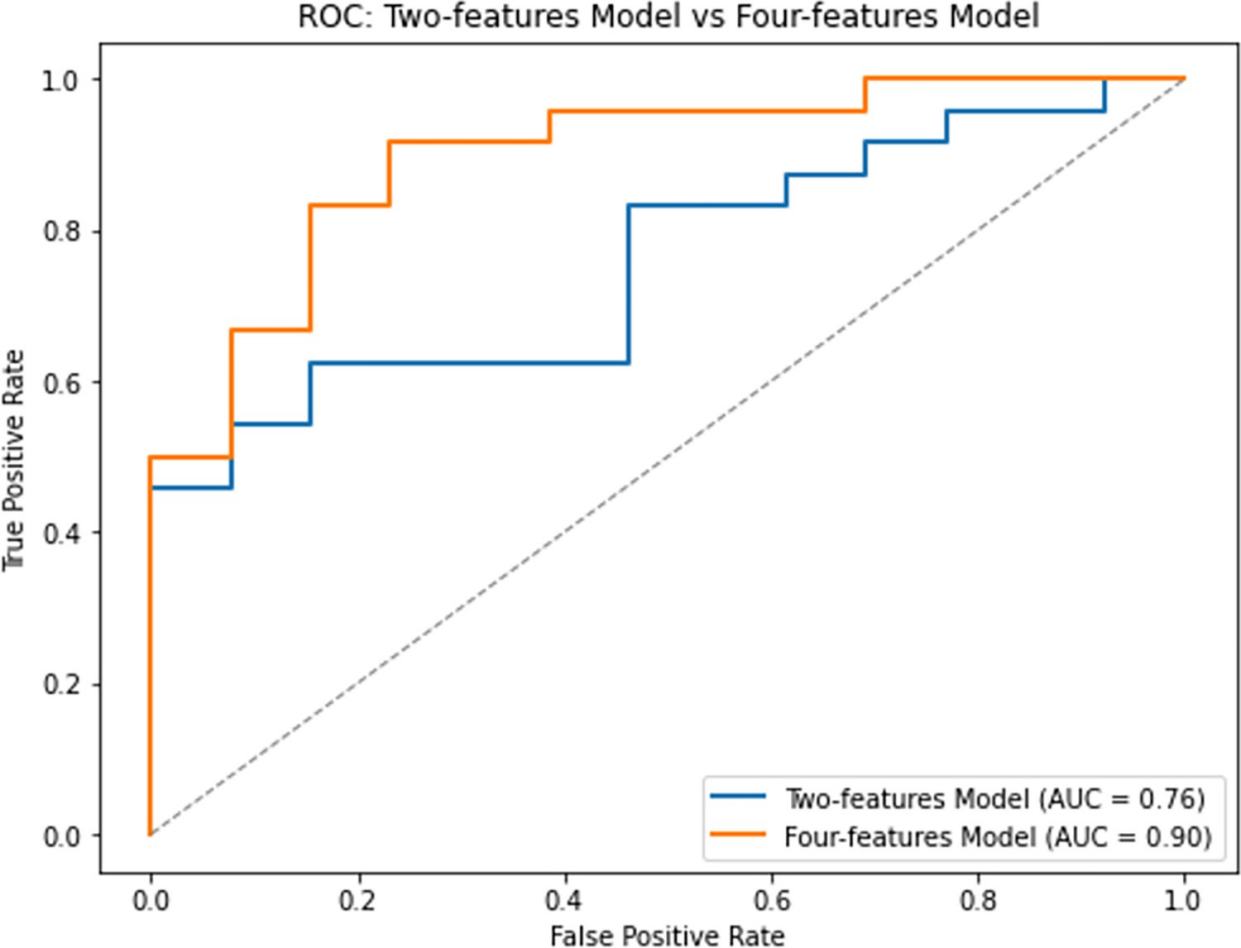
Comparison of the receiver operating characteristic (ROC) curves of the two-features model and with four-features model. The curve is derived from the sensitivity and specificity index, i.e., the rate of samples classified correctly in the positive and negative classes.

Our results show that both neural networks trained on sequential data demonstrated encouraging accuracy in classifying motor trajectories using both the two-features model trained on raw dataset and the four-features model. The results show that the four-features model has an overall higher distribution of accuracy than the two-features model (Fig 6). This suggests that the four-features model generally provides more accurate predictions than the two-features model. This performance difference between the two models can be also seen in Fig 7, where the Receiver Operating Characteristic (ROC) curve is shown. Specifically, we observe that the two-features model trained with 2 features, i.e. x and y coordinates, has an AUC (Area Under the Curve) of 0.76 (sensitivity = 0.83, specificity = 0.54). In contrast, the four-features model trained with 4 features has an AUC of 0.90 (sensitivity = 0.92, specificity = 0.77).

**Fig 8:**
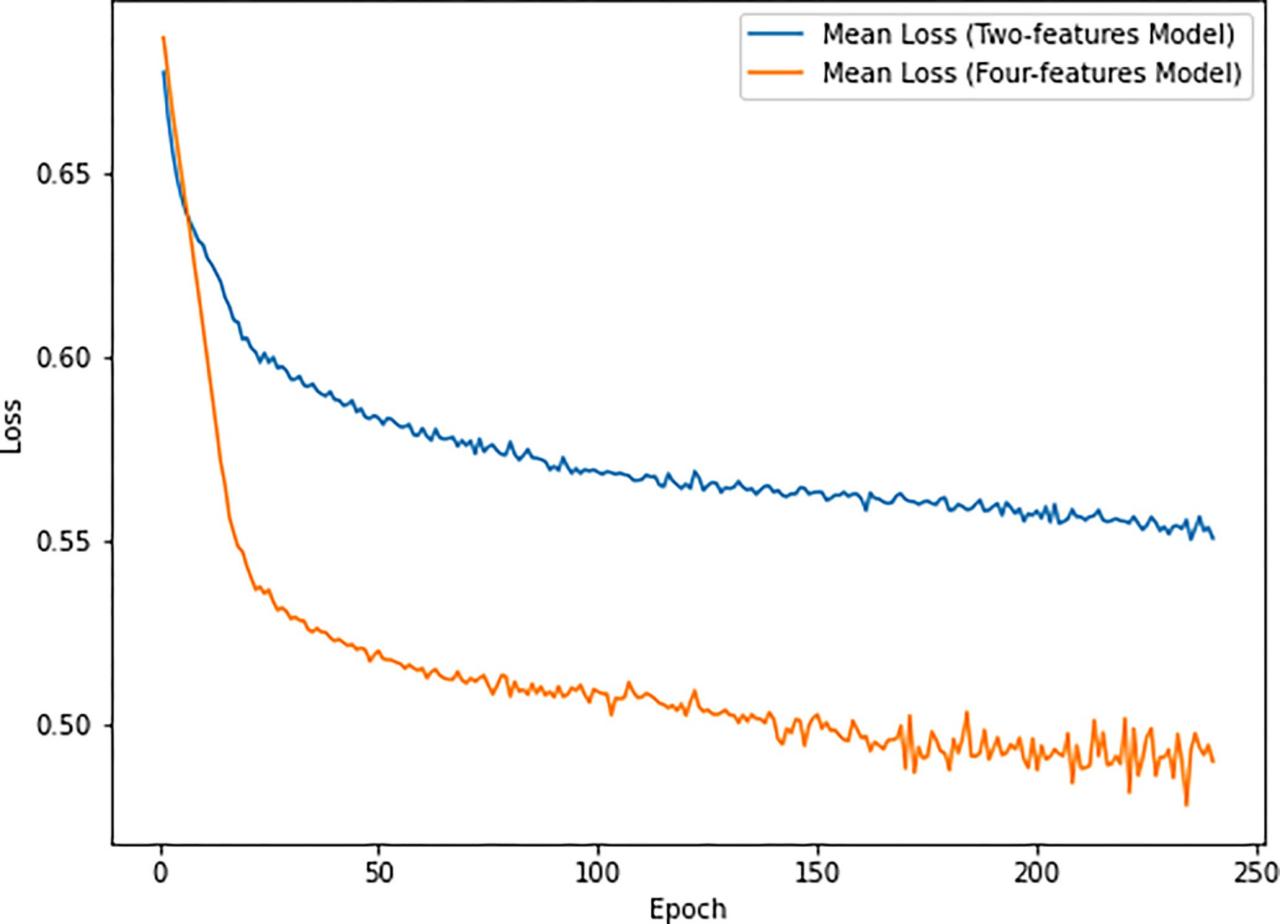
Curve of loss. Average loss during the training process for each model configuration (2 features and 4 features) based on the cross-validation results.

To evaluate the predictive ability of the two models in classifying motor trajectories, we considered the average loss (loss) in the four folds of cross validation, during the training process within the first 240 epochs. The results, shown in Fig 8, indicated that both models progressively reduced the loss. However, the two-features model trained with raw dataset has a higher average loss within the 240 epochs than the four-features model (x and y coordinates, velocity, and acceleration), which has better predictive ability than the two-features model. To examine the relationship between the average acceleration of each trajectory and the predictions of our models, we utilized Pearson’s correlation coefficient, on 932 samples.

**Fig 9:**
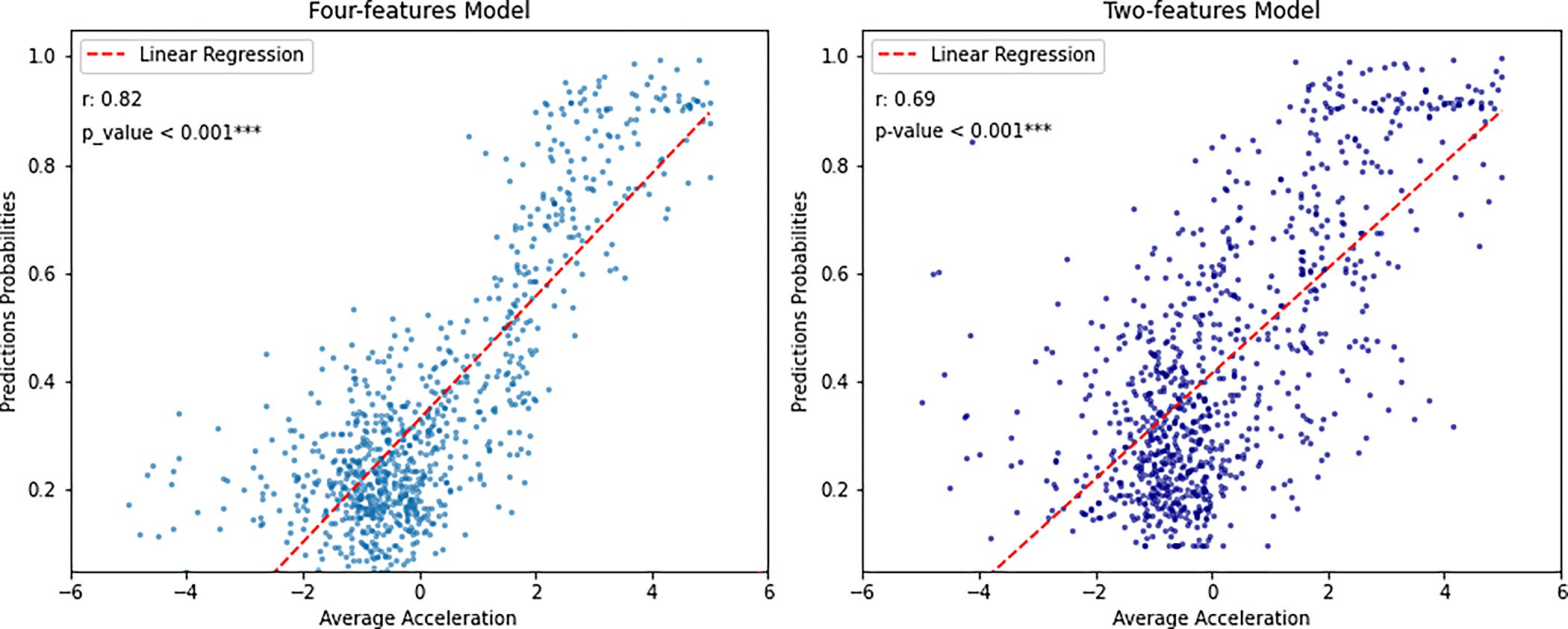
Relationship between average acceleration, expressed in cm^2/s, and model predictions. This graph displays the relationship between average acceleration of trajectories (on the x-axis) and ML model predictions (on the y-axis) for two different approaches: The two-features model (in dark blue) and the four-features model (in blue). Furthermore, the graph has been augmented to display both the p-values and Pearson’s correlation coefficients (r), associated with each model, providing a statistical measure of the strength and direction of the linear relationship between average acceleration and model predictions.

Results obtained on simulated trajectories, depicted in Fig 9, considering an acceleration range between -5 cm^2/s and +5 cm^2/s, showed a moderately positive correlation (r = 0.69) between the average acceleration of the trajectories and the predictions. On the other hand, the four-features model reached a stronger relationship between average trajectory acceleration and predictions (r = 0.82). For both model, the statistical significance of correlation is further emphasized by a p-value < 0.00. As the average acceleration value increases, the model is more likely to predict belonging to the ASD group.

## Discussion

For many decades, even though neither a cause nor a cure is known, the assessment and diagnosis of ASD have relied primarily on observable behavioral variables, often with qualitative assessment. In recent years, there has been a significant effort by researchers and health professionals to develop quantitative and objective measures to address the challenges associated with autism diagnosis [41]. Recognition of motor abnormalities as precursors of future deficits in social interactions and language has made possible the development of increasingly sophisticated tools based on quantitative data [30,31,42–44].

However, most of these studies require the analysis of a great amount of features to obtain a complete picture of the target behavior. In line with the suggestions of Zhao et al. (2021) [45] and Milano et al. (2023) [31], that highlighted the growing need to identify globally optimal features for the diagnosis of ASD, to reduce data processing, high consumption of computational resources and, most importantly, to avoid the inclusion of features that are not essential for diagnosis. Thus, the goal of researchers in this field is to identify relevant features for ASD classification and find the most ecological model to compute them. In line with the requirement to employ the most relevant and essential features for the diagnosis of the disorder, we decided to implement and test a model trained on sequential points of the trajectories obtained in a "drag and drop" task. The aim was to focus exclusively on the raw data, avoiding the use of a wide range of additional complex and often superfluous features.

The LSTM neural network was chosen for its capabilities to analyze temporal sequences and automatically detect patterns and salient features in sequential data, without the need for pre-processing of the data, reducing computational complexity, and the risk of including nonessential features.

Furthermore, in order to better evaluate the performance of a two-features model, exclusively composed of raw data, we compared it with a four-features model, which included, in addition to the sequential points, trajectory information, such as point-by-point accelerations and velocities. Our results showed that, the performance of the four-features model was better than the two-features model, namely, 0.90 and 0.76, respectively (Fig 7). Despite this, the two-features model reached a reasonable classification accuracy.

These findings suggest that the exclusive use of raw data, although it has been largely unexplored by researchers, may offer new opportunities to explore raw motion without adding value inferences to such behavior. Analyzing raw data certainly allows for a more objective way to analyze these behaviors.

Another important goal of our study was to investigate the influence of acceleration on the classification process between groups of ASD and TD, as previous research suggested that acceleration might be a significant factor in this classification [31]. So, after implementing and training the models, we tested them on simulated trajectories and the result confirmed that acceleration represents a core component of the prediction of autism, confirming the results from existing literature [30,31]. By the means of simulated trajectories, we observed that acceleration was positively correlated with autism diagnosis in both models. Specifically, the correlation coefficient was higher in the four-features model. This model was better at capturing and explaining variation based on the average acceleration of trajectories. This could be since the four-features model was trained by considering not only the sequential points of the trajectory, but also the point-by-point velocities and accelerations, while the two-features model had to make a greater effort in extracting more information about the data for classification.

These outcomes shed light on how to enhance the classification process by minimizing data processing and using as few features as possible. However, our findings did not answer the question concerning the effectiveness of using raw data in reducing computational workload.

In order to critically analyze our findings, it is important to take into account the limitations of the study.

Although the study shows promising results, further validations in real-world contexts and across larger populations could help confirm the effectiveness of the models. Additionally, cross-validation with other diagnostic tools and longitudinal studies could provide a deeper understanding of the models’ predictive capacity over time.

In fact, one potential limitation of this study might be the small sample size and the exclusive use of a tablet device as a motion tracking tool. A more extensive and advanced data collection approach could lead to a more accurate analysis, even when solely relying on raw data. The sample should be expanded and observed longitudinally. Different types of measures could be coordinated to obtain more detailed information, such as manual and ocular movement data, to inform us about hand-eye movement patterns.

These considerations and our results pave the way for future studies, but without losing sight that when working with children’s populations, especially children with disabilities such as autism, it is crucial to create a relaxing environment for accurate behavioral assessment. Adopting gamified approaches, such as the use of game software on tablets, not only provides immediate, automatic, and quantitative data, but also creates an ecological and friendly assessment setting [35].

Another potential limitation is the noisiness of the data. Collecting data from real-world settings, exposes to the risk of noise more than pre-processed data. Raw data recorded directly from sensors or acquisition devices may contain various types of noise due to unintended movements, natural variability in motor skills, or technical disturbances during data collection [46,47].

Addressing these limitations requires a broader and more diverse data collection method, to better handle the noise and obtain a more complete representation of the variations in motor behavior. These efforts can help improve the robustness and validity of future research in this field.
